# From n + n to 2n + n: Unconventional Chromosome Inheritance and Stable Retention of the Entire *Erianthus rockii* Genome in Sugarcane Hybrids

**DOI:** 10.3390/plants15121792

**Published:** 2026-06-10

**Authors:** Xueting Li, Yirong Guo, Zhejun Guo, Nannan Zhang, Jiayun Wu, Zuhu Deng, Qinnan Wang

**Affiliations:** 1 Guangdong Sugarcane Genetic Improvement Engineering Center, Institute of Nanfan and Seed Industry, Guangdong Academy of Sciences, Guangzhou 510316, China; lxt19910226@163.com (X.L.);; 2 National Engineering Research Center for Sugarcane, Fujian Agriculture and Forestry University, Fuzhou 350002, China

**Keywords:** *Erianthus rockii*, sugarcane, GISH, chromosome inheritance, wide hybridization, unreduced gametes, introgression breeding

## Abstract

Hybridization between sugarcane (*Saccharum* spp.) and its wild relative *Erianthus rockii* offers a promising route to broadening the narrow genetic base of modern cultivars, but the authenticity and chromosome inheritance patterns of such hybrids remain poorly understood. In this study, we combined molecular marker (tetra-primer ARMS-PCR) and cytogenetic (genomic in situ hybridization, GISH) approaches to verify hybridity and track chromosome transmission in 24 F_1_ and 12 BC_1_ progeny. The F_1_ hybrids exhibited a strict n + n transmission pattern, receiving exactly 15 chromosomes from *E. rockii*. When F_1_ plants were used as the male parent in backcrosses, no BC_1_ seeds were obtained due to complete pollen sterility. Remarkably, when F_1_ plants served as the female parent, all 12 BC_1_ clones retained the entire set of 15 *E. rockii* chromosomes intact, following an unconventional 2n + n pattern. These findings reveal a strong parent-of-origin effect and, for the first time, demonstrate that the whole *E. rockii* chromosome complement can be stably transmitted into backcross progeny without loss or recombination. This opens a direct route for introgressing complete wild genomes into sugarcane breeding lines, preserving complex polygenic traits and guiding rational crossing strategies.

## 1. Introduction

*Saccharum* spp. is an important sugar crop, accounting for 80% of the world’s sugar production [1]. Sugarcane is a complex high-ploidy crop (2n = 40~128). Due to long-term hybridization between cultivated species, the genetic basis of sugarcane is narrow. This narrow genetic base limits yield gains and increases vulnerability to biotic and abiotic stresses [2]. In order to increase production and expand the genetic base, breeders have crossed *Saccharum* spp. with other related genera to obtain disease- and stress-resistant materials and contribute to sugarcane cultivars with increased biomass, disease resistance and tillering ability. Related species targeted for crossing have included *Erianthus* Michanx. sect. *Ripidium* Henrard., *Sclerostachya* (Hach) A. Camus, *Narenga* Bor and *Miscanthus* Anderss., which are all members of the so-called “*Saccharum* complex” [3].

In recent years, *Erianthus rockii* Keng. [4,5], *Tripidium arundinaceus* [6,7], *Narenga* Bor. [8] and other related genera materials have been used in sugarcane breeding programs, greatly increasing the genetic basis of sugarcane resistance. Among these, *E. rockii* is a wild relative species in the genus *Erianthus* closely related to sugarcane, mainly distributed within China in Sichuan, Yunnan, and Tibet, and across the Indochina Peninsula. *E. rockii* is allotetraploid, and the common *E. rockii* has 30 chromosomes (2n = 30) [9,10]. It harbors valuable agronomic traits such as rust resistance, drought tolerance, cold tolerance, and strong tillering ability, which are highly desirable for sugarcane improvement [10]. Prior to the development of molecular markers, hybrid identity was merely inferred from morphological traits, and the authenticity of offspring was only inferred based on morphological characteristics and agronomic data.

Recent advances in sugarcane genomics have greatly improved our understanding of polyploid architecture. A polyploid genome assembly of the modern sugarcane cultivar R570 revealed that ~77% of the assembly originated from *Saccharum officinarum* L. and ~22% from *Saccharum spontaneum* L., with extensive interspecific recombinant chromosomes [11]. Another study deciphered the highly allo-autopolyploid modern sugarcane genome, providing insights into very recent allopolyploidization events in *Saccharum* [12]. A near-complete genome assembly of allotetraploid *E. rockii* showed that the *Saccharum* complex originated from chromosome fusion and polyploidization events tracing back to a diploid ancestor approximately 5.1 million years ago, with sub-genomic differences in centromere dynamics, epigenetic variation, and three-dimensional genome reorganization underlying concerted diploidization [9]. Furthermore, the investigation of fusion chromosome formation and centromere inactivation in two allotetraploid species (*E. rockii* and *N. porphyrocoma*) demonstrated that the 10 fusion chromosomes originated through nested chromosome fusion, end-to-end fusion, and centromere misdivision, with breakpoints consistently located in proximal regions near ancestral centromeres and accompanied by centromere inactivation [13]. Together, these genomic and cytogenetic advances provide a foundational framework for interpreting the chromosome inheritance mechanisms observed in the present study, particularly regarding the stable retention of intact *E. rockii* chromosomes across generations.

With the development of sequencing technology and DNA molecular markers, the genome of *E. rockii* has been deciphered, and many identification methods have been developed [9]. Recent research findings, including cloning analysis of ITS sequences from different sugarcane-related species and molecular markers, were designed to identify the relationship of *E. rockii* [14]. In order to distinguish *E. rockii*, we utilized a tetra-primer amplification refractory mutation system PCR (tetra-primer ARMS PCR); this method involves designing primers at the SNP sites of nrDNA-ITS and amplifying bands of different sizes through PCR to detect the SNP. Tetra-primer ARMS PCR is rapid and efficient. Tetra-primer ARMS PCR technology has been widely used to identify various germplasm genotypes of sugarcane, rice, wheat, and other crops [15,16,17].

We used genomic in situ hybridization (GISH) to identify hybrid progeny and characterize the chromosome inheritance and composition of the hybrid progeny [18,19,20]. GISH has been widely applied in karyotype analysis of maize, potato, peanut and other plant species to characterize chromosome composition. D’Hont et al. adopted genomic DNA as probes to clarify ploidy variations among *Saccharum* species [21]. Compared with molecular markers, GISH allows direct visualization of parental chromosomes in hybrid backgrounds, providing unambiguous evidence of hybridization and enabling precise tracking of chromosome inheritance across generations. This technique is particularly valuable in polyploid species such as sugarcane, where conventional genetic analysis is complicated by complex chromosome pairing and segregation pattern [22].

In the process of interspecific sugarcane hybridization, chromosome inheritance frequently deviates from the canonical n + n Mendelian inheritance mode. In the progeny of *S. officinarum* and *S. spontaneum*, the chromosome inheritance pattern of F_1_ is 2n + n, F_1_ backcrosses with *S. officinarum* as the male parent, BC_1_ was 2n + n, and BC_2_ becomes n + n [20,23]. In the progeny of *S. officinarum* and *T. arundinaceus*, the inheritance of chromosome in F_1_ was n + n, F_1_ backcrosses with *S. officinarum* as the female parent, BC_1_ was 2n + n or ≥ 2n + n, while in BC_2_, it was n + n mode [7,18,19]. The mechanism underlying non-Mendelian chromosome inheritance in the process of hybridization may be due to their distant relationship, chromosome incompatibility or abnormal meiosis in the process of meiosis [24], or chromosome doubling during zygote fusion.

To date there have been relatively few reports on the identification of hybrid progeny of *E. rockii*, and fewer still on the chromosome inheritance mode of hybrid progenies [10,25]. We used GISH to identify the authenticity of F_1_ and BC_1_ hybrids between sugarcane and *E. rockii*, and studied the genetic pattern of progenies according to the number of chromosomes. The creation of the germplasm of true *E. rockii* progeny was conducive to the expansion and introduction of resistance genes in sugarcane breeding, accelerating the utilization efficiency of wild germplasm resources, and the analysis of chromosome inheritance mode has a guiding role in the utilization of *E. rockii* in sugarcane, including the selection of appropriate parental combinations and backcross strategies.

## 2. Results

### 2.1. Identification of Suspected E. rockii Materials

#### 2.1.1. Tetra-Primer ARMS PCR Identification of *E. rockii* Materials

**Figure 1:**
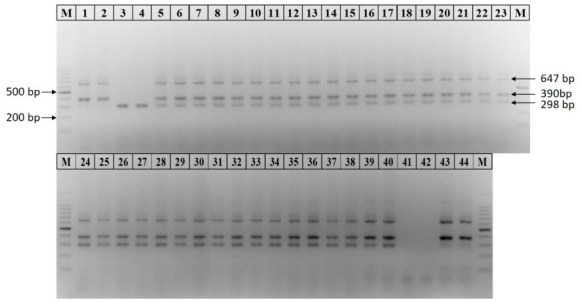
Gel electrophoresis analysis of tetra-primer ARMS-PCR product. Note: 100 bp Marker; 1–2: Badila; 3–4: *E. rockii*; 5–28: F_1_; 29–40: BC_1_; 41–42: H_2_O; 43–44: Sugarcane cultivar XTT22.

A tetra-primer ARMS-PCR molecular identification system was developed based on the nrDNA-ITS region to discriminate *E. rockii*-specific alleles from those of sugarcane. As shown in Figure 1, the *E. rockii* parent clones amplified a specific 298 bp DNA fragment, whereas the *S. officinarum* ‘Badila’ parent yielded two bands of 647 bp and 390 bp. All 24 F_1_ hybrids and 12 BC_1_ progeny simultaneously displayed all three diagnostic bands (647 bp, 390 bp and 298 bp), confirming their biparental origin. No false positives were observed in negative controls. This rapid, co-dominant marker system enables early-generation screening of true hybrids, eliminating the need for time-consuming field evaluations.

#### 2.1.2. Genomic In Situ Hybridization

**Figure 2:**
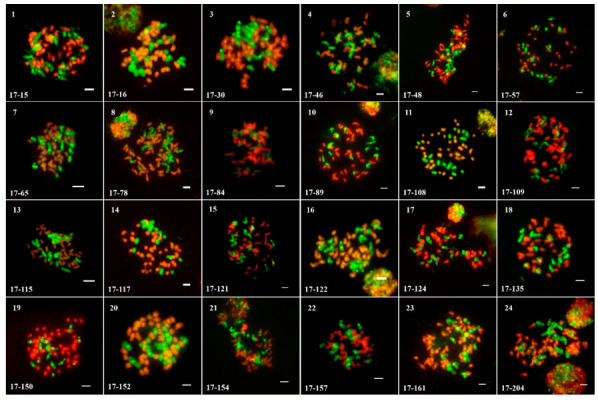
Genomic in situ hybridization of F_1_ progenies. Note: The green color represents the chromosome of *E. rockii*, while the red or yellow-red color represents the chromosome of Badila. 1–24 is the hybrid F_1_ of Badila and *E. rockii*. The scale is 5 μm.

**Figure 3:**
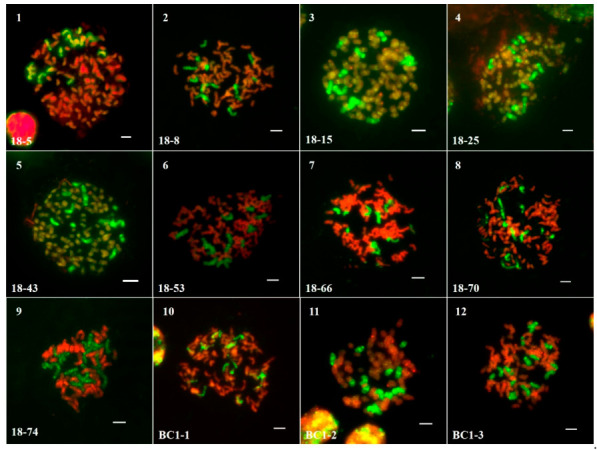
Genomic in situ hybridization of BC_1_ progenies. Note: The green color represents the chromosome of *E. rockii*, while the red or yellow-red color represents the chromosome of Badila. 1–12 is the hybrid BC_1_ of Badila and *E. rockii*. The scale is 5 μm.

GISH was performed on root tip metaphase chromosomes to directly visualize the chromosomal contribution of each parent. *E. rockii* chromosomes were labeled in green, while the *S. officinarum* ‘Badila’ chromosomes appeared red or yellow-red (Figure 2 and Figure 3). The wild parent *E. rockii* used in this study had a somatic chromosome number of 2n = 30.

In all 24 F_1_ clones (Figure 2), exactly 15 green-labeled (*E. rockii*) and 40 red-labeled ‘Badila’ chromosomes were observed, demonstrating a strict n + n transmission pattern. Notably, all *E. rockii* chromosomes appeared structurally intact, with no detectable intercalary recombination signals. The complete, unrecombined inheritance of half the wild genome in F_1_ preserves parental linkage blocks, facilitating the subsequent evaluation of *E. rockii*-derived traits.

Due to F_1_ pollen sterility, the F_1_ hybrid can only be used as the maternal parent for hybridization and can produce hybrid progenies. When F_1_ hybrids were used as the female parent in backcrosses with sugarcane cultivars (male parent), 12 BC_1_ clones were successfully obtained. GISH analysis of these BC_1_ clones (Figure 3) revealed that each BC_1_ individual also contained 15 intact *E. rockii* chromosomes (green), despite the backcrossing process. This retention of the complete wild chromosome set corresponds to a 2n + n transmission pattern. As in the F_1_ generation, no loss or structural rearrangement of *E. rockii* chromosomes was observed in any BC_1_ clone.

Collectively, the GISH results demonstrate that: F_1_ hybrids follow an n + n chromosome inheritance pattern. BC_1_ progeny derived from female F_1_ parents exhibit a 2n + n pattern with stable retention of all 15 *E. rockii* chromosomes. The wild chromosomes are transmitted intact without immediate recombination or loss, offering a promising pathway for introgressing *E. rockii* traits into sugarcane breeding lines.

## 3. Discussion

This study provides significant insights into the chromosomal inheritance patterns in hybrids between sugarcane (*S. officinarum*) and its wild relative *E. rockii*. By applying tetra-primer ARMS PCR and GISH, this study reveals atypical chromosome inheritance patterns in these distant hybrids, showing inheritance patterns significantly different from canonical Mendelian rules [8]. The stable retention of *E. rockii* chromosomes, however, also brings a serious limitation: the introgression of all negative or undesired genes located on a given chromosome, in addition to any positive gene meant to be introgressed. This phenomenon is known among breeders as ‘genetic drag’. The combination of molecular marker screening and cytogenetic visualization proved to be a powerful strategy for hybrid verification and chromosome tracking, which can be readily applied to other wide hybridization programs within the *Saccharum* complex [26,27].

Regarding the inheritance pattern of 2n + n chromosomes in offspring, two major pathways have been proposed to explain unreduced gamete formation: 2n gametes may result from nondisjunction of homologous chromosomes during meiosis I (first division restitution, FDR) or nondisjunction of sister chromatids in meiosis II (second division restitution, SDR) [7]. Additionally, when F_1_ serves as the female parent, the unconventional 2n + n inheritance pattern was observed in the BC_1_ progeny. The 2n + n inheritance pattern probably results from cytokinesis failure or pre-meiotic endoreduplication [28,29]. Alternatively, pre-meiotic endoreduplication, failure of cytokinesis, or spindle abnormalities may also contribute to unreduced gamete formation. These mechanisms warrant further investigation using molecular cytogenetic approaches such as fluorescence in situ hybridization with centromere-specific probes or the analysis of microsatellite marker segregation patterns in unreduced gametes [30].

The conservation of the *E. rockii* chromosome (15 chromosomes) across both F_1_ and BC_1_ generations is particularly noteworthy. This chromosomal stability suggests the existence of mechanisms that actively maintain the wild species genome in the hybrid background, potentially through preferential chromosome pairing or selective retention of *E. rockii* chromosomes. Such a finding has significant implications for trait introgression breeding, as it indicates that entire chromosomal segments from wild relatives can be transferred to cultivated species without immediate fragmentation or recombination. This is in contrast to many other interspecific hybrids where rapid chromosome elimination or rearrangement occurs, such as in wheat × barley hybrids [31]. The stable retention of wild chromosomes may facilitate the transfer of complex polygenic traits (e.g., drought tolerance, rust resistance) that are controlled by multiple genes distributed across the *E. rockii* genome.

Chromosome evolution in eukaryotes involves complex cycling events: the number of chromosomes changes, reflecting cycles of chromosome number increase (polyploidy and centric fissions) and decrease (chromosome fusions); during this process, chromosome rearrangement occurs, including nested chromosome fusions (NCFs) and end-to-end fusions (EEFs) [32]. These structural features, involving breakpoints near centromeric regions and the inactivation of one ancestral centromere per fusion event, may contribute to the unusual meiotic behavior observed in the hybrids [33,34]. To place our findings in context, Table 1 compares chromosome transmission patterns across different wide hybrids within the *Saccharum* complex such as variable patterns in sugarcane hybrids with *Erianthus arundinaceus* or the typical 2n + n patterns observed in early-generation hybrids with *Saccharum spontaneum* [35,36], which often undergo progressive “genome dilution” in advanced backcrosses—a contrast to the persistent transmission seen with *E. rockii* [37]. This diversity underscores the importance of species-specific cytological characterization before committing to large-scale introgression programs.

This study offers significant insights into chromosomal evolution and genome stabilization processes in distant plant hybrids. The persistent transmission of intact *E. rockii* chromosomes across generations points to genetic mechanisms that preserve chromosomal integrity, possibly through preferential pairing or selection against recombinant chromosomes [11]. The directional hybridization barrier, where success is achieved only when the F_1_ serves as the female parent, further indicates the involvement of cytoplasmic–nuclear incompatibilities or genomic imprinting effects that differentially affect development based on parental genome origin. For example, mitochondrial or chloroplast genomes from the female parent may interact with nuclear genes from *E. rockii* to influence pollen fertility or embryo development. This hypothesis could be tested by reciprocal crossing experiments using different cytoplasmic backgrounds. These results are of particular significance in the context of current research progress in sugarcane genomics, including the sequencing of modern cultivars and the resolution of fusion chromosome structures in *E. rockii*, which provide a contextual framework for the observed cytological patterns of complex polyploid architecture in sugarcane and its relatives [12,13]. Compared with other wild relatives, *E. rockii* exhibits a unique pattern where the full wild genome complement is stably maintained across at least two generations when the F_1_ is used as the female parent. This stability may be attributed to the specific genomic structure of *E. rockii*, including its allotetraploid nature and fused chromosome architecture, as well as possible genetic incompatibilities that prevent the recombination or loss of *E. rockii* chromosomes.

*E. rockii* possesses valuable agronomic traits such as rust resistance, drought and cold tolerance, and strong tillering ability, which are highly desirable for sugarcane improvement. The documented unconventional inheritance pattern presents both challenges and opportunities. The consistent transmission of the entire *E. rockii* chromosome complement suggests a potential pathway for whole chromosome introgression of desirable multigenic trait complexes without immediate recombination and dilution, potentially accelerating the transfer of wild traits into cultivated sugarcane. Furthermore, the finding that F_1_ hybrids are more successful as the female parent provides practical guidance for optimizing breeding strategies. The developed tetra-primer ARMS PCR protocol also enables reliable early identification of true hybrids, saving time and resources required for field evaluation.

Based on the findings, promising future research directions include detailed cytological and transcriptomic analyses of meiosis in F_1_ hybrids to elucidate the mechanisms of unreduced gamete formation, producing and characterizing advanced backcross generations to track inheritance pattern persistence, comprehensive phenotypic evaluation for agronomically important traits, and the application of genomic selection (GS) and genome-wide association studies (GWAS) to identify chromosomal regions linked to desirable traits [38,39]. Expanded crossing programs and investigations into the physiological basis of the directional hybridization barrier could further enhance the efficiency of wide hybridization in sugarcane improvement.

Our results provide three actionable insights for breeding programs aiming to utilize *E. rockii*. Maternal use of F_1_ is essential, because F_1_ plants are male sterile but produce functional unreduced female gametes; backcrosses must use F_1_ as the female parent. This simple rule prevents wasted effort on reciprocal crosses. Whole-genome introgression is feasible in one backcross. The stable transmission of all 15 *E. rockii* chromosomes into BC_1_ means that breeders can obtain progeny carrying the complete wild genome without immediate recombination or loss. This is particularly valuable for complex polygenic traits (e.g., drought tolerance, rust resistance) that are likely distributed across multiple *E. rockii* chromosomes. Subsequent backcrosses may gradually reduce wild chromosomes. While BC_1_ retains the full set, further backcrossing (BC_2_, BC_3_) with sugarcane as the recurrent parent would be expected to progressively eliminate *E. rockii* chromosomes through meiotic loss unless selection is applied. Therefore, a strategy of early phenotypic selection for desirable traits, followed by targeted backcrossing with marker assistance, is recommended.

### Limitations and Future Directions

Our study has four main limitations that define priorities for future research.

First, the mechanism of unreduced female gamete formation remains unknown. Is it due to first division restitution (FDR), second division restitution (SDR), or pre-meiotic endoreduplication? Using centromere-specific markers or Oligo-FISH on unreduced gametes would distinguish these possibilities and could reveal whether the phenomenon is genetically tractable.

Second, although *E. rockii* chromosomes are stably transmitted intact in BC_1_, no recombination between wild and cultivated chromosomes was observed. For many agronomic traits, some level of recombination is eventually needed to break linkage with undesirable alleles.

Third, we did not evaluate the agronomic performance of BC_1_ clones. It is unknown whether the presence of all 15 *E. rockii* chromosomes confers beneficial traits, deleterious effects, or both. Field trials measuring yield, sugar content, disease resistance, and stress tolerance in replicated trials are urgently needed. These data will determine whether the effort of whole-genome introgression is justified or whether selection for specific chromosomes is preferable.

Fourth, whole-chromosome introgression will only allow the transfer of wild genes that are present on chromosomes carrying no negative/undesirable genes. Such a situation is expected to be very rare, representing a serious obstacle to bringing *E. rockii* genes into sugarcane. Furthermore, the fact that F_1_ hybrids can only be used as female parents inevitably results in the introgression of cytoplasmic DNA from *E. rockii*, which may or may not be desirable depending on the specific genetic background and breeding objectives. It will also result in the substitution of cytoplasmic genes from *S. officinarum*, which is present in all sugarcane cultivars currently grown in the world, by cytoplasmic genes from *E. rockii*, with unknown effects.

## 4. Materials and Methods

### 4.1. Plant Material

The plant materials were provided and preserved at the Hainan Sugarcane Breeding Center, Sanya, China. F_1_ hybrids were obtained by crossing with *S. officinarum* ‘Badila’ (as female) with *E. rockii* (as male). BC_1_ progenies were generated via backcrossing with F_1_ plants as the female parent and sugarcane cultivars (XTT22, DZ93-88, YN83-168) as the male parent. We collected 24 F_1_ clones and 12 BC_1_ clones for experimentation. All plant materials were maintained under standard field conditions with regular irrigation and pest management. Please refer to Table 2 for details on parentage and generation information.

### 4.2. Methods

#### 4.2.1. Tetra-Primer ARMS PCR Procedure

High-quality genomic DNA was isolated from Badila (*S. officinarum*) and *E. rockii* using the CTAB method [40]. The tetra-primer Amplification Refractory Mutation System PCR (ARMS-PCR) was designed based on the nrDNA-ITS region [14]. The amplification was performed using the following primers: FO1 (5′-TCCGTAGGTGAACCTGCGG-3′), RO1 (5′-CGGGTAGTCCCGACTGACCT-3′), FI1 (5′-AAGTTGCGCCCGAGGCCTTTTA-3′), and RI1 (5′-AGGCAGACGTGCCCTCGTCCG-3′). The PCR reaction mixture was prepared as described in Table 3. The amplification protocol consisted of an initial denaturation step at 95 °C for 5 min; followed by 6 cycles of denaturation at 95 °C for 30 s, annealing at 64 °C for 30 s (with a decrease of 1 °C per cycle), and extension at 72 °C for 20 s; then 28 cycles of denaturation at 95 °C for 30 s, annealing at 58 °C for 30 s, and extension at 72 °C for 30 s; with a final extension at 72 °C for 5 min. PCR products were separated by electrophoresis on a 2.0% agarose gel for detection.

#### 4.2.2. Preparation and Screening of Mid-Term Cells

Please refer to the root tip cultivation method [41]. The root tip meristem was excised and incubated in 0.075 mol·L^−1^ KCl solution or water at 37 °C for 30 min. Root tip samples were digested with mixed enzymatic solution containing 1% pectolyase Y23 (Yakult, Minato, Japan), 2% pectinase (Solarbio, Beijing, China), 2% Cellulase “Onozuka” RS (Yakult, Minato, Japan), and 4% Cellulase “Onozuka” R-10 (Yakult, Minato, Japan) in a volume ratio of 2:1:1:1, followed by incubation at 37 °C for approximately 4 h. After enzymatic digestion, the residual enzyme solution was carefully removed, and the tissue was washed with ddH_2_O. Subsequently, ddH_2_O was added again and allowed to stand for 30 min. The ddH_2_O was then aspirated, and freshly prepared Carnoy’s fixative was introduced. The apical meristem cells were dispersed by gentle pipetting. A 1 μL aliquot of cell suspension was loaded onto a clean pre-cooled slide, followed by the addition of 10 μL of fixative. Prior to complete evaporation of the fixative, 10 μL of acetic acid was applied to facilitate chromosome spreading. The slide was air-dried at room temperature. Metaphase chromosome spreads were screened using a microscope (Axio Imager A2, ZEISS, Oberkochen, Germany), and slides with well-dispersed chromosomes were selected for further analysis.

#### 4.2.3. Preparation of Genomic Probes

The probe was labeled with biotin using Badila (*S. officinarum*) DNA, and with digoxigenin using *E. rockii* DNA, obtained by enzyme digestion and the specific preparation reaction. Probe size was checked by agarose gel electrophoresis, with optimal fragment sizes ranging from 200–500 bp. The hybridization and detection were performed according to standard protocols [42], with post-hybridization washes under stable conditions. The mix was shown in Table 4. The total number of cells observed for each material exceeds 20.

## 5. Conclusions

This study demonstrates that sugarcane × *E. rockii* F_1_ hybrids follow an n + n pattern, and when used as the female parent, they transmit the entire set of 15 *E. rockii* chromosomes intact to BC_1_ via a 2n + n mechanism. The complete pollen sterility of F_1_ males makes the maternal backcross the only viable route. These findings provide a clear breeding strategy—use F_1_ as female—and show that whole-genome introgression of *E. rockii* is achievable in one backcross without recombination. The intact, stable retention of wild chromosomes positions *E. rockii* as a strategic resource to broaden the narrow genetic base of sugarcane. However, we recognize that this interpretation is speculative, because it would be valid only if chromosome recombination between the two species can be achieved. These findings provide a theoretical basis and practical guidance for utilizing *E. rockii* germplasm in sugarcane introgression breeding, and contribute to a deeper understanding of chromosome inheritance mechanisms in distant hybridization within the *Saccharum* complex. Future efforts should focus on inducing recombination between wild and cultivated chromosomes and evaluating the agronomic performance of advanced backcross populations.

## Figures and Tables

**Table 1:** Chromosomal inheritance patterns in distant hybridization of sugarcane.

Hybrid Combination	F_1_ Pattern	BC_1_ Pattern	Fertility Issues
Sugarcane × *E. rockii*	n + n	n + 2n (paternal F_1_)	F_1_ pollen sterile
Sugarcane × *E. arundinaceus*	n + n	2n + n or ≥2n + n	F_1_ pollen sterile
Sugarcane × *E. fulvus* [35]	n + n	2n + n (F_2_)	F_1_ pollen sterile
Sugarcane × *S. spontaneum* [36]	2n + n	2n + n	Variable

**Table 2:** Parental and progeny information of F_1_ and BC_1_ clones.

Number	Generation	Progeny Name	Female Parent	Male Parent
1	F_1_	ERK17-15	Badila	*E. rockii*
2	F_1_	ERK17-16	Badila	*E. rockii*
3	F_1_	ERK17-30	Badila	*E. rockii*
4	F_1_	ERK17-46	Badila	*E. rockii*
5	F_1_	ERK17-48	Badila	*E. rockii*
6	F_1_	ERK17-57	Badila	*E. rockii*
7	F_1_	ERK17-65	Badila	*E. rockii*
8	F_1_	ERK17-78	Badila	*E. rockii*
9	F_1_	ERK17-84	Badila	*E. rockii*
10	F_1_	ERK17-89	Badila	*E. rockii*
11	F_1_	ERK17-108	Badila	*E. rockii*
12	F_1_	ERK17-109	Badila	*E. rockii*
13	F_1_	ERK17-115	Badila	*E. rockii*
14	F_1_	ERK17-117	Badila	*E. rockii*
15	F_1_	ERK17-121	Badila	*E. rockii*
16	F_1_	ERK17-122	Badila	*E. rockii*
17	F_1_	ERK17-124	Badila	*E. rockii*
18	F_1_	ERK17-135	Badila	*E. rockii*
19	F_1_	ERK17-150	Badila	*E. rockii*
20	F_1_	ERK17-152	Badila	*E. rockii*
21	F_1_	ERK17-154	Badila	*E. rockii*
22	F_1_	ERK17-157	Badila	*E. rockii*
23	F_1_	ERK17-161	Badila	*E. rockii*
24	F_1_	ERK17-204	Badila	*E. rockii*
25	BC_1_	ERK18-5	ERK17-66	XTT22
26	BC_1_	ERK18-8	ERK17-108	XTT22
27	BC_1_	ERK18-15	ERK17-84	XTT22
28	BC_1_	ERK18-25	ERK17-84	XTT22
29	BC_1_	ERK18-43	ERK17-141	XTT22
30	BC_1_	ERK18-53	ERK17-134	DZ93-88
31	BC_1_	ERK18-66	ERK17-108	DZ93-88
32	BC_1_	ERK18-70	ERK17-108	DZ93-88
33	BC_1_	ERK18-74	ERK17-108	DZ93-88
34	BC_1_	BC1-1	ERK17-108	DZ93-88
35	BC_1_	BC1-2	ERK17-89	YN83-168
36	BC_1_	BC1-3	ERK17-154	XTT22

**Table 3:** Tetra-primer ARMS PCR mixtures.

Components	Volume (μL)
ddH_2_O	11.9 − X
10 × Ex Taq buffer	2
dNTP (2.5 mM each)	2
FO1 (10 μM)	1
RO1 (10 μM)	1
FI1 (10 μM)	1
RI1 (10 μM)	1
gDNA (50 ng)	X
Ex Taq (5 U/μL)	0.1
Total volume	20

**Table 4:** Hybrid probe preparation system.

Experimental Reagent	Volume (μL)
0.1 mM dNTP mix with digoxigenin or biotin	25
10 × DNase I buffer	5
0.0005 U/μL DNase I	5
Polymerase I	5
gDNA (5 μg)	X
ddH_2_O	5 − X
Total	50

## Data Availability

The materials generated in this study are available from the corresponding author upon request.

## References

[1] Tew T.L., Cobill R.M. (2008). Genetic improvement of sugarcane (*Saccharum* spp.) as an energy crop. Genetic Improvement of Bioenergy Crops.

[2] Nayak S.N., Song J., Villa A., Pathak B., Ayala-Silva T., Yang X., Todd J., Glynn N.C., Kuhn D.N., Glaz B. (2014). Promoting utilization of *Saccharum* spp. genetic resources through genetic diversity analysis and core collection construction. PLoS ONE.

[3] Amalraj V.A., Balasundaram N. (2006). On the Taxonomy of the Members of ‘*Saccharum* Complex’. Genet. Resour. Crop Evol..

[4] Cai Q., Aitken K.S., Fan Y.H., Piperidis G., Jackson P., Mcintyre C.L. (2005). A preliminary assessment of the genetic relationship between Erianthus rockii and the “*Saccharum* complex” using microsatellite (SSR) and AFLP markers. Plant Sci..

[5] Aitken K., Li J., Wang L., Qing C., Fan Y.H., Jackson P. (2007). Characterization of intergeneric hybrids of *Erianthus rockii* and *Saccharum* using molecular markers. Crop Evol. Genet. Resour..

[6] Deng Z.-H., Zhang M.-Q., Lin W.-L., Cheng F., Zhang C.-M., Li Y.-C., Lai L.-P., Lin Y.-Q., Chen R.-K. (2010). Analysis of Disequilibrium Hybridization in Hybrid and Backcross Progenies of *Saccharum officinarum* × *Erianthus arundinaceus*. J. Integr. Agric..

[7] Wu J., Huang Y., Lin Y., Fu C., Liu S., Deng Z., Li Q., Huang Z., Chen R., Zhang M. (2014). Unexpected Inheritance Pattern of *Erianthus arundinaceus* Chromosomes in the Intergeneric Progeny between *Saccharum* spp. and *Erianthus arundinaceus*. Front. Plant Sci..

[8] Chang H., Wang Q., Qiu Y., Qin Y., Li X., Wu Q., He W., Guo Y., Zhang W., Chen J. (2020). Production, Identification and Characterization of *Erianthus rockii* × *Narenga porphyrocoma* Intergeneric Hybrids as a New Germplasm for Sugarcane Breeding and Genetic Research. Sugar Tech.

[9] Wang B., Zhang Z., Qi Y., Wei Y., Mei J., Liu H., Wang Y., Fang Y., Hua X., Ding H. (2025). Near-complete genome assembly of allotetraploid *Erianthus rockii* reveals unique chromosome evolution and lineage-divergence trajectories in the *Saccharum* complex. Plant Commun..

[10] Xiuqin L., Xin L., Xinlong L., Jun M., Qiuyan Z., Hongbo L., Zhonglei L., Xujuan L., Chaohua X., Chunjia L. (2017). Analysis of Intergeneric Hybrids (F_1_, BC_1_) of *Saccharum* × *Erianthus rockii* using Morphology and GISH Technique. Mol. Plant Breed..

[11] Healey A.L., Garsmeur O., Lovell J.T., Shengquiang S., Sreedasyam A., Jenkins J., Plott C.B., Piperidis N., Pompidor N., Llaca V. (2024). The complex polyploid genome architecture of sugarcane. Nature.

[12] Zhang J., Qi Y., Hua X., Wang Y., Wang B., Qi Y., Huang Y., Yu Z., Gao R., Zhang Y. (2025). The highly allo-autopolyploid modern sugarcane genome and very recent allopolyploidization in *Saccharum*. Nat. Genet..

[13] Yu Z., Huang Y., Yu X., Deng Z., Chai J., Liu J., Gong Z., Yao W., Zhang J., Zhang M. (2025). Formation and centromere inactivation of fusion chromosomes in two allotetraploid species from the *Saccharum* complex. New Phytol..

[14] Wang G., Wang J., Zhang W., Xu H., Liang Q., Qin Y., Wu Q., Wu J., Fu C., Zhou F. (2025). Identification and validation of intergeneric hybrids between *Saccharum officinarum* and *Erianthus rockii* using molecular and cytogenetic tools. PLoS ONE.

[15] Yang S., Li X., Huang F., Huang Y., Liu X., Wu J., Wang Q., Deng Z., Chen R., Zhang M. (2018). A new method based on SNP of nrDNA-ITS to identify *Saccharum spontaneum* and its progeny in the genus *Saccharum*. PLoS ONE.

[16] Ouyang J., Zhu Z., Guan Y., Huang Q., Huang T., Zang S., Pan C. (2026). Novel Wx Gene Functional Markers for High-Resistant Starch Rice Breeding. Genes.

[17] Saxesena R.R., Mishra V.K., Chand R., Kumar U., Chowdhury A.K., Bhati J., Budhlakoti N., Joshi A.K. (2022). SNP Discovery Using BSR-Seq Approach for Spot Blotch Resistance in Wheat (*Triticum aestivum* L.), an Essential Crop for Food Security. Front. Genet..

[18] Huang Y., Wu J., Wang P., Lin Y., Fu C., Deng Z., Wang Q., Li Q., Chen R., Zhang M. (2015). Characterization of Chromosome Inheritance of the Intergeneric BC_2_ and BC_3_ Progeny between *Saccharum* spp. and *Erianthus arundinaceus*. PLoS ONE.

[19] Yang S., Zeng K., Chen K., Wu J., Wang Q., Li X., Deng Z., Huang Y., Huang F., Chen R. (2019). Chromosome transmission in BC_4_ progenies of intergeneric hybrids between *Saccharum* spp. and *Erianthus arundinaceus* (Retz.) Jeswiet. Sci. Rep..

[20] Yu F., Wang P., Li X., Huang Y., Wang Q., Luo L., Jing Y., Liu X., Deng Z., Wu J. (2018). Characterization of chromosome composition of sugarcane in nobilization by using genomic in situ hybridization. Mol. Cytogenet..

[21] D’Hont A., Ison D., Alix K., Roux C., Glaszmann J.C. (1998). Determination of basic chromosome numbers in the genus *Saccharum* by physical mapping of ribosomal RNA genes. Genome.

[22] Piperidis N., D’Hont A. (2020). Sugarcane genome architecture decrypted with chromosome-specific oligo probes. Plant J. Cell Mol. Biol..

[23] Nair M.K. (1972). Cytogenetics of *Saccharum officinarum* L., *Saccharum spontaneum* L. and *S. officinarum* × *S. spontaneum* hybrids II. Cytologia.

[24] Li X., Huang F., Chai J., Wang Q., Yu F., Huang Y., Wu J., Wang Q., Xu L., Zhang M. (2021). Chromosome behavior during meiosis in pollen mother cells from *Saccharum officinarum* × *Erianthus arundinaceus* F_1_ hybrids. BMC Plant Biol..

[25] Kui L., Di Y.-N., Majeed A., Yang Z.-J., Chen J.-W., He L.-L., Wang X.-H., Liu L.-F., Qian Z.-F., Zeng D. (2022). Evaluation of genome size and phylogenetic relationships of the *Saccharum* complex species. 3 Biotech.

[26] Meng Z., Han J., Lin Y., Zhao Y., Lin Q., Ma X., Wang J., Zhang M., Zhang L., Yang Q. (2019). Characterization of a *Saccharum spontaneum* with a basic chromosome number of x = 10 provides new insights on genome evolution in genus *Saccharum*. Theor. Appl. Genet..

[27] Xu S., Wang J., Shang H., Huang Y., Yao W., Chen B., Zhang M. (2018). Transcriptomic characterization and potential marker development of contrasting sugarcane cultivars. Sci. Rep..

[28] D’Hont A., Grivet L., Feldmann P., Rao S., Berding N., Glaszmann J.C. (1996). Characterisation of the double genome structure of modern sugarcane cultivars (*Saccharum* spp.) by molecular cytogenetics. Mol. Gen. Genet..

[29] Pachakkil B., Terajima Y., Ohmido N., Ebina M., Irei S., Hayashi H., Takagi H. (2019). Cytogenetic and agronomic characterization of intergeneric hybrids between *Saccharum* spp. hybrid and *Erianthus arundinaceus*. Sci. Rep..

[30] De Storme N., Geelen D. (2013). Sexual polyploidization in plants—Cytological mechanisms and molecular regulation. New Phytol..

[31] Malysheva L., Sjakste T., Matzk F., Röder M., Ganal M. (2003). Molecular cytogenetic analysis of wheat-barley hybrids using genomic in situ hybridization and barley microsatellite markers. Genome.

[32] Lysak M.A. (2022). Celebrating Mendel, McClintock, and Darlington: On end-to-end chromosome fusions and nested chromosome fusions. Plant Cell.

[33] Ran M., Yu B., Cheng C., Li X., Guo Y., Zhao L., Zan F., Lin X., Hou X., Zhao Y. (2025). Oligo-FISH-Based Analysis of the Mechanisms Underlying Chromosome Number Variation in *Saccharum spontaneum*. Int. J. Mol. Sci..

[34] Chai J., Xue L., Lei J., Yao W., Zhang M., Deng Z., Yu F. (2023). All nonhomologous chromosomes and rearrangements in *Saccharum officinarum* × *Saccharum spontaneum* allopolyploids identified by oligo-based painting. Front. Plant Sci..

[35] Wang X., Li F., He L., Lou H., Yang Q., He S. (2014). Chromosomal Transmission in F2BC1Hybrid Progenies between Sugarcane and *Erianthus fulvus*. Agricultural Biotechnology.

[36] Ren S., Zhao L., Tao L.A., Zhang Y., Zan F., Lu X., Zhao Y., Zhang J., Liu J. (2025). Analysis of Genetic Diversity in Polymers of *Saccharum spontaneum* L. and Their Hybrid Progenies. Agronomy.

[37] Cai Q., Aitken K.S., Fan Y.H., Piperidis G., Liu X.L., Mcintyre C.L., Huang X.Q., Jackson P. (2012). Assessment of the genetic diversity in a collection of *Erianthus arundinaceus*. Genet. Resour. Crop Evol..

[38] Sandhu K.S., Shiv A., Kaur G., Meena M.R., Raja A.K., Vengavasi K., Mall A.K., Kumar S., Singh P.K., Singh J. (2022). Integrated Approach in Genomic Selection to Accelerate Genetic Gain in Sugarcane. Plants.

[39] O’Connell A., Deo J., Deomano E., Wei X., Jackson P., Aitken K.S., Manimekalai R., Mohanraj K., Hemaprabha G., Ram B. (2022). Combining genomic selection with genome-wide association analysis identified a large-effect QTL and improved selection for red rot resistance in sugarcane. Front. Plant Sci..

[40] Porebski S., Bailey L.G., Baum B.R. (1997). Modification of a CTAB DNA extraction protocol for plants containing high polysaccharide and polyphenol components. Plant Mol. Biol. Report..

[41] Li X., Guo Y., Huang F., Wang Q., Chai J., Yu F., Wu J., Zhang M., Deng Z. (2022). Authenticity Identification of *Saccharum* officinarum and *Saccharum spontaneum* Germplasm Materials. Agronomy.

[42] Piperidis N. (2014). GISH: Resolving interspecific and intergeneric hybrids. Methods Mol. Biol..

